# Ectopic adrenal gland tissue in the left ovary of an elderly woman: a case report

**DOI:** 10.11604/pamj.2021.40.181.31064

**Published:** 2021-11-24

**Authors:** Seon Mi Lee, Jong Chul Baek, Ji Eun Park, Hyen Chul Jo, Hyun Min Koh

**Affiliations:** 1 Department of Obstetrics and Gynecology, Gyeongsang National University Changwon Hospital, Gyeongsang National University, School of Medicine, Changwon, Korea,; 2 Department of Pathology, Gyeongsang National University Changwon Hospital, Changwon, Korea

**Keywords:** Ectopic adrenal gland, pelvic ovary, elderly woman, case report

## Abstract

Ectopic adrenal gland in the ovary is very rare case, and even more rarer in older women. We reported a case of ectopic adrenal tissue as an incidental finding in left ovary from a 68-year-old woman. She presented with bearing down sensation due to uterine prolapse for 5 years. Upon physical examination, uterine prolapse grade III, cystocele, and rectocele were observed. Ultrasonography findings showed 0.69 cm intramural myoma, and no specific findings were found in the bilateral adnexae. She underwent a total laparoscopic hysterectomy, bilateral salpingo-oophorectomy, and anterior-posterior repair. The final pathologic diagnosis of the case was ectopic adrenal gland tissue in the left ovary and uterine leiomyoma. No eventful reactions were observed during hospitalization and after discharge. Although ectopic adrenal gland rarely occurs in elderly women and in the pelvic ovaries, it has a risk of neoplastic transformation and accompanying germ cell tumor and sex cord tumor. Hence, if the ectopic adrenal gland tissue is suspected during surgery, the tissue should be removed. Additionally, by closely examining the contralateral ovary, determining whether other lesions are suspected is necessary. If the other lesions including germ cell tumor or sex cord tumor are suspected, a biopsy of the contralateral ovarian tissue should be performed. Thus, gynecologists must have knowledge about ectopic adrenal gland tissues.

## Introduction

Ectopic adrenal gland tissue is induced by small fragments of the adrenal cortex originating from the ectoderm during the course of invasion into the central vein during adrenal gland development, and was first reported by Morgani in 1740 [1]. According to the embryological process of this tissue, it is mainly observed in the spermatogenic cord region and the retroperitoneum, but it is rarely observed in the ovary, broad ligament, testis, stomach, and liver. The incidence rates according to the location of the ectopic adrenal gland tissue reported in other papers are presented as follows: celiac axis area (32%), broad ligament (23%), kidney (0.1%-6%), and adnexa of the testis (7.5%) [2]. This tissue is mainly observed in male children with a probability of approximately 50%, and it is rarely observed in adults, especially in women, because it becomes atrophied by the normal function of the adrenal glands in adulthood [3]. It is usually asymptomatic, and most cases are diagnosed incidentally as a result of groin surgical exploration in children and other surgical treatments for other purposes in adults [3]. Furthermore, an ectopic adrenal gland tissue tends to be observed more on the right side than on the left, and it is rare on both sides [4]. We report a rare case of ectopic adrenal gland tissue observed in the left ovary of a 68-year-old female.

## Patient and observation

**Patient information:** a 68-year-old multiparous (gravida 2, para 2) woman presented with a bearing down sensation due to uterine prolapse grade III that lasted for 5 years. At the time the patient was first diagnosed with uterine prolapse, she had a follow-up observation at a local hospital after a pessary was inserted, but she visited this hospital for surgical treatment. Past medical history of hypertension was reported. She was taking medications due to hypertension, and her blood pressure was well controlled.

**Clinical findings:** as a result of physical examination, there was no palpable mass in the lower abdomen area, and there was no tenderness at all. As a result of gynecology examination, there were no specific findings other than grade III uterine prolapse.

**Diagnostic assessment:** a transvaginal sonographic evaluation revealed a 0.69 cm intramural-type uterine myoma, and no other lesions were observed on both adnexa.

**Therapeutic intervention:** the symptoms of the patient´s uterine prolapse were causing discomfort in her daily life. As she was post-menopausal, her ovaries were no longer producing female hormones. For these reasons, the patient agreed to a prophylactic bilateral salpingo-oophorectomy during hysterectomy. Although no ovarian lesions were present, neither of the ovaries were functional and there was a risk that lesions such as ovarian malignant masses may develop later. Therefore, the patient underwent laparoscopic hysterectomy with prophylactic bilateral salpingo-oophorectomy as well as anterior and posterior repairs. The surgically removed uterus, bilateral ovaries, and fallopian tubes were sent to the pathology department for histological analysis.

**Figure 1:**
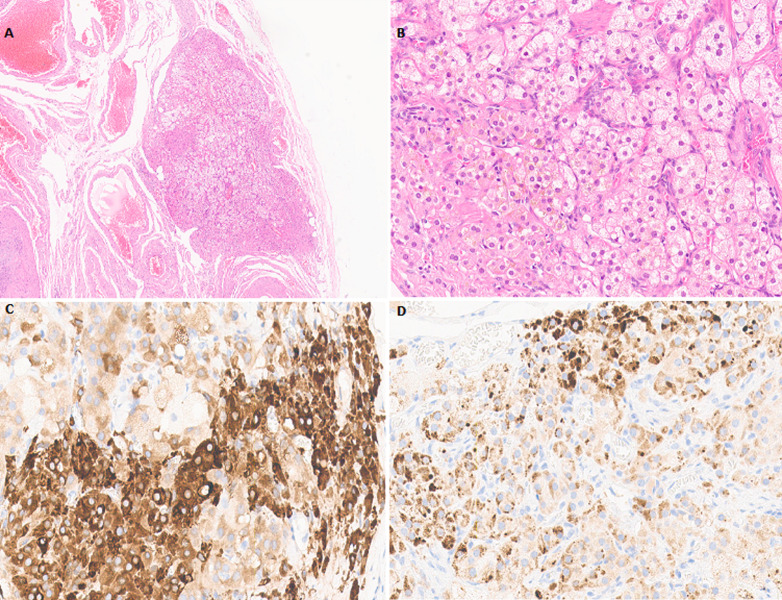
histologic and immunohistochemical findings in the left ovary; (A) a hematoxylin-eosin stained image of an ectopic adrenal tissue in the left ovary; microscopic examination shows pale cells with spongy-appearing cytoplasm and polygonal cells with acidophilic granular cytoplasm, which is reminiscent of zona fasciculata and zona reticularis of the adrenal cortex ((A) x20, (B) x100); (C, D) immunohistochemical stains are positive for inhibin-A ((C) x200) and Melan-A ((D) x200)

On section, the cut surface showed intramural leiomyoma and both adnexa were unremarkable, grossly. Microscopic examination revealed 0.3x0.2x0.2 cm sized ectopic adrenal tissue in the left ovary. The tissue consisted of pale cells with spongy-appearing cytoplasm and polygonal cells with acidophilic granular cytoplasm, which is reminiscent of zona fasciculata and zona reticularis of the adrenal cortex (Figure 1A, Figure 1B). The ectopic tissue was reactive for inhibin-A and Melan-A, which favored the diagnosis of ectopic adrenal tissue (Figure 1C, Figure 1D). The final pathological diagnosis was leiomyoma and ectopic adrenal tissue on the left ovary. She was discharged on the seventh day after the surgery.

**Follow-up and outcome of interventions:** the patient presented with no complications six months after surgery.

**Informed consent:** the patient consented to the publication of a case report containing her clinical data; because her case was rare, and she wanted to be helpful in the treatment of such a case in the future.

## Discussion

Although no ovarian lesions were found by our on the preoperative ultrasound, we chose to perform a prophylactic bilateral salpingo-oophorectomy during the patient´s hysterectomy based on consideration of the cost-benefit balance. The risk of ovarian cancer increases with age, such that the prevalence rises from 20 per 100,000 among women aged 50 years to 40 per 100,000 women aged 70 years [5]. Ovarian preservation is recommended in women aged < 65 with a low risk of ovarian cancer because the benefits it affords, such as reducing the risks of cardiovascular disease and fractures, are greater than the risk of ovarian cancer [5]. However, the same study recommends that women aged > 65 years decide whether to undergo resection or preserve their ovaries based on sufficient explanation of the risk of ovarian cancer [5]. In this case, the patient consented to bilateral salpingo-oophorectomy during her hysterectomy. This decision was based on the lack of benefits to post-menopausal preservation of the ovaries and the benefit of reduced risk of ovarian cancer afforded by the procedure. Therefore, we performed a laparoscopic hysterectomy and a bilateral salpingo-oophorectomy with anterior and posterior repair. As a result, ectopic adrenal gland tissue was found.

Ectopic adrenal gland tissue is located in a site other than the normal location of the adrenal gland. The development of adrenal glands occurred during the fourth and fifth weeks of gestation and comprises an adrenal cortex originating from the mesoderm and an adrenal medulla originating from the ectoderm [1, 6]. During fetal development, the medullary tissue invades the cortex along the central vein, and small fragments from the defect migrated to the abnormal adrenal site becoming the ectopic adrenal gland [1]. The tissue is seen grossly as a yellow nodule assembling to the adipose tissue on the surface, and microscopically, it comprises three zones that make up the adrenal tissue. The first zone is located just below the capsule and contains small clusters and short trabeculae called zona glomerulosa, the second fasciculate forms a broad band composed of large cells, and the cytoplasm is characterized by the presence of lipid-containing vacuoles. The third zone, or the reticularis, is composed of cells smaller than the fasciculate of the second layer, and this cytoplasm is lipid-free and is composed of granular and acidophilic cells. As the ectopic adrenal gland moves further than the existing adrenal gland location, only the cortex tends to remain among the components of the adrenal gland [6, 7]. As a result of immunohistochemical analysis, positive for Melan-A and Inhibin-A and negative for AE1/AE3 is characteristic of this tissue [6]. According to the embryological theory described above, this tissue is frequently observed not only in the spermatic cord region but also in the celiac axis area, broad ligament, adnexa of testes, kidney, lung, and intracranial cavity [2].

Ectopic adrenal gland tissue can also be observed in other mammals, but in most humans, it is found in childhood with a 50% occurrence, and the average age of onset is approximately 5.8 years. Because it becomes atrophied with age, the incidence rate drops sharply with a probability of 1% in adults, and it is particularly rare to be observed in adult women [3]. According to the papers published so far, the location of ectopic adrenal tissue is usually higher on the right side than on the left, and this tissue is rarely found on both sides [4]. Because most ectopic adrenal gland tissues present in childhood or adulthood are asymptomatic, they are observed incidentally as a result of histopathological examination after surgical treatment. However, rarely, cases of corticotropin-independent Cushing´s syndrome in which an ectopic adrenal gland tissue performs an endocrine function and causes symptoms including hypertension, central obesity, and abdominal striae have been reported [8]. Moreover, a case of neoplastic transformation of ectopic adrenal gland tissue was reported by Goren *et al*. [9]. Because germ cells and adrenal cells migrate together during the embryonic process, the coexistence of germ cell tumors with ectopic adrenal tissues may occur. A case of coexistence of the ectopic adrenal gland in the left ovary and granulosa cell tumor in the right ovary was reported by Yasar *et al*. [10].

## Conclusion

An ectopic adrenal gland is a very rare in elderly women, and is often diagnosed incidentally following surgical treatment and histological examination. However, surgical removal of the tissue is necessary because of metabolic and neoplastic complications. Furthermore, carefully checking whether there are any suspicious findings of a malignant ovarian tumor in the contralateral ovary is needed, and a biopsy should be performed if necessary.
